# An Agave Counting Methodology Based on Mathematical Morphology and Images Acquired through Unmanned Aerial Vehicles

**DOI:** 10.3390/s20216247

**Published:** 2020-11-02

**Authors:** Gabriela Calvario, Teresa E. Alarcón, Oscar Dalmau, Basilio Sierra, Carmen Hernandez

**Affiliations:** 1 Department of Electronics, Systems, and Informatics, ITESO—The Jesuit University of Guadalajara, Tlaquepaque, Jalisco 45604, Mexico; gabriela.calvario@iteso.mx; 2 Departamento de Ciencias Computacionales e Ingenierías, Centro Universitario de los Valles, Ameca, Jalisco 46600, Mexico; 3 Centro de Investigación en Matemáticas, Guanajuato 36023, Mexico; dalmau@cimat.mx; 4 Departamento de Ciencias de la Computación e Inteligencia Artificial, Universidad del País Vasco UPV/EHU, 20018 Donostia-San Sebastián, Spain; b.sierra@ehu.es (B.S.); mamen.hernandez@ehu.es (C.H.); 5 Centre for the Research and Technology of Agro-Environmental and Biological Sciences, CITAB, Universidade de Trás-os-Montes e Alto Douro, UTAD, 5000-801 Vila Real, Portugal

**Keywords:** precision agriculture, UAV, data mining, computer vision, geomatics, crop monitoring

## Abstract

Blue agave is an important commercial crop in Mexico, and it is the main source of the traditional mexican beverage known as tequila. The variety of blue agave crop known as Tequilana Weber is a crucial element for tequila agribusiness and the agricultural economy in Mexico. The number of agave plants in the field is one of the main parameters for estimating production of tequila. In this manuscript, we describe a mathematical morphology-based algorithm that addresses the agave automatic counting task. The proposed methodology was applied to a set of real images collected using an Unmanned Aerial Vehicle equipped with a digital Red-Green-Blue (RGB) camera. The number of plants automatically identified in the collected images was compared to the number of plants counted by hand. Accuracy of the proposed algorithm depended on the size heterogeneity of plants in the field and illumination. Accuracy ranged from 0.8309 to 0.9806, and performance of the proposed algorithm was satisfactory.

## 1. Introduction

**Figure 1:**
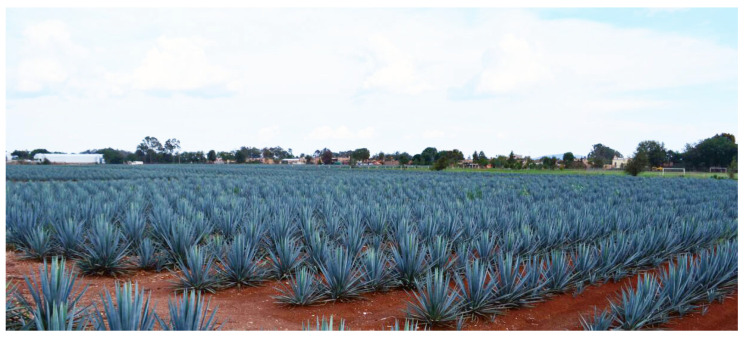
Image of agave plantation: the image is from [2].

Blue agave is a succulent plant that grows in arid and warm areas [1]. Blue agave (see Figure 1) is a native Mexican plant, and it is the principal source for production of the traditional Mexican beverage known as tequila. Tequila producers need to estimate the yield of agave plantation in order to plan and predict production of the drink. The number of agave plants is one of the parameters that determines the yield of plantation; hence, the elaboration of tequila requires precise control and monitoring of the number of agave plants. Despite the existence of several modern techniques in Mexican agriculture, monitoring of the agave crop is mainly done manually with farm tools, which requires great effort to achieve good counting precision. According to our research, there are three main reasons that explain the predominant use of manual counting of agave plants in Mexico: the lack of information about the usefulness of image processing methods in agriculture, the lack of affordable and innovative techniques for agave monitoring, and the loss of jobs that this type of technology brings.

**Figure 2:**
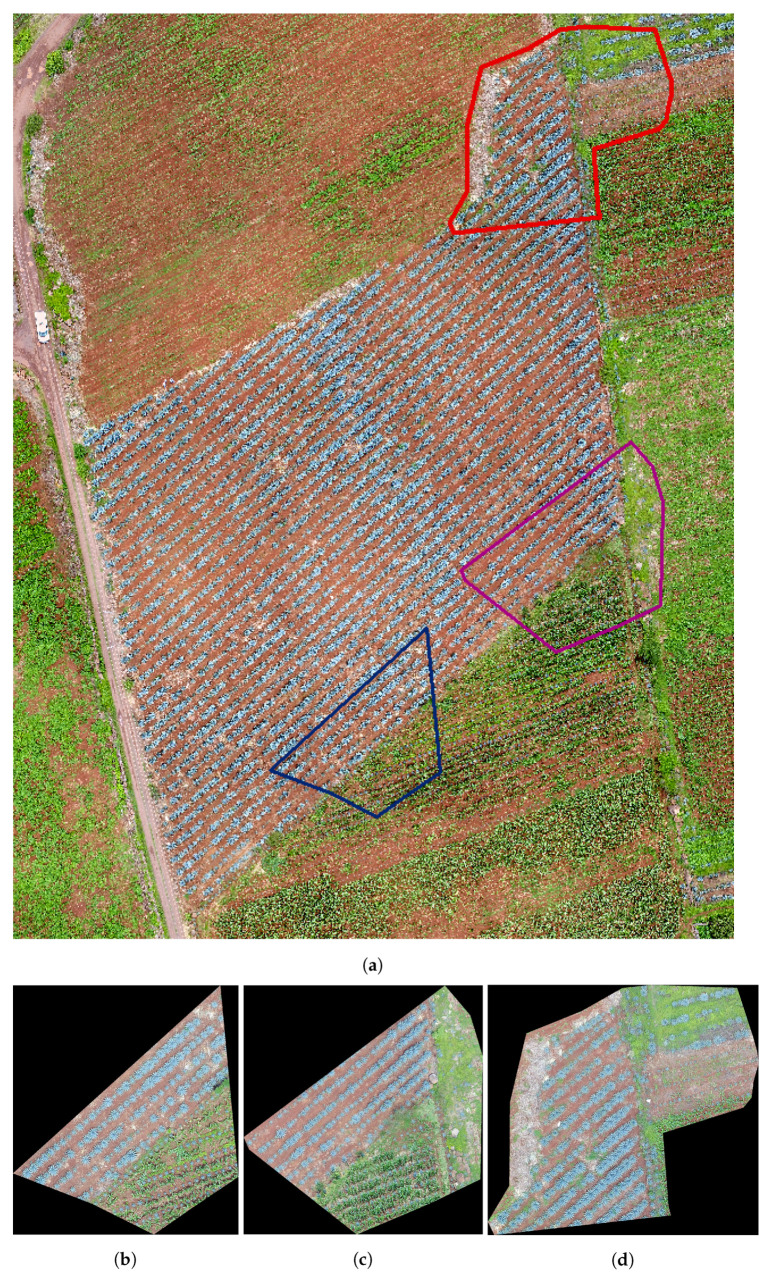
Images of Field 1 obtained with an Unmanned Aerial Vehicle (UAV): (**a**) Field 1 with some marked regions that are studied in this work, (**b**) the region in blue, (**c**) the region in purple, and (**d**) the region in red.

Although there are algorithms for counting plants [3,4,5,6], to the best of our knowledge, there is no reliable algorithm for agave counting. One limitation when applying state-of-the-art algorithms for agave plant counting is the overlap among plants that can be seen in Figure 1 and Figure 2. In this manuscript, we present an algorithm for counting agave plants which is very useful in monitoring agave plantation. The proposal is very important due to it allowing improvements in the estimation of Tequila production. Our algorithm is based on the agave segmentation methodology proposed by Calvario et al. [2], in which the scene was acquired through Unmanned Aerial Vehicle (UAV) and segmented by using k-means [7]. One drawback of the previous work is that it does not solve the overlap problem presented in agave plantation, which makes this algorithm useless for direct agave counting. The main contribution of this work is the elaboration of a methodology for agave counting in order to better control and monitor agave yield. We describe the implementation of agave counting algorithm based on the theory of mathematical morphology (MM) [8,9]. One advantage of MM is the computational efficiency, additionally, it does not require large image datasets to estimate the parameters of algorithms.

## 2. Related Work

Remote sensing and digital image processing have contributed to precision agriculture. In particular, Unmanned Aerial Vehicles (UAVs) and image processing have been used in agriculture to better monitor and control the yield parameters of different crops. UAV can provide a very high image resolution even in terms of millimeters depending on the altitude of the flight and resolution of the camera [10]. This is very relevant for management and monitoring of the crop without being in direct contact and with a low cost [2]. On the other hand, digital image processing allows automatic or semiautomatic analysis of images and extracts useful information for farmers. In the literature, we can find methods that combine UAV technique with computer vision and artificial intelligence that allows for the extraction of useful information for agriculture such as segmentation of regions corresponding to a specific crop. Meanwhile, there are other methods that directly address the counting problem.

In [10], the authors evaluated the performance of six different vegetation indices: Color index of vegetation (CIVE) [11], Excess green (ExG) [12], Excess green minus excess red (ExGR) [13], Woebbecke Index [12], Normalized green-red difference index (NGRDI) [14], and Vegetativen (VEG) [15] for studying wheat crop images acquired with a digital Red-Green-Blue (RGB) camera installed in UAV. In order to discriminate the wheat crop, they used Otsu’s method [16]. The authors assessed the accuracy, and spatial and temporal consistency of the mentioned indices and conclude that ExG and VEG contribute with the highest classification accuracy. The investigators in [17] proposed an OBIA [18] algorithm based on Otsu’s threshold method and thoroughly studied how different parameters of a multiresolution segmentation algorithm during the segmentation step affect the classification of vegetation coverage. In their research, they considered images of vegetation indices ExG [12] and the Normalized Difference Vegetation Index (NDVI) [19] to enhance the information about vegetation. The authors in [17] test the proposal for vegetation detection in UAV images acquired over three different crops: wheat, sunflower, and maize. In [20], the authors carried out research using images acquired from two UAV platforms: vertical taking off landing multi-rotor quadcopter and fixed wing UAV. In their proposal, they combined the spectral and spatial information. The purpose of that study was to detect crop tomato regions and to classify tree crown. In particular, they used Bayesian information criterion [21] to set the optimal number of clusters for a given image. Subsequently, they applied k-means [7] and Expectation Maximization (EM) [22] algorithms for clustering using spectral information. To improve clustering, they incorporated spatial information through an agglomerative approach based on majority voting method.

On the other hand, Gnädinger and Schmidhalter [3] carried out research to demonstrate the efficiency of the UAV and image analysis as a possible technique for counting plants. In their study, they analyzed four different maize cultivars and the images were acquired by means of UAVs. In order to discriminate the ground cover from the cultivars, they used a thresholding technique. On average, they achieved an error ≤ 5% between visually and digitally counted plants. The authors in [23] considered 10 different vegetation indices and image classification from UAV-based RGB multispectral images for estimating the flower number in two different oilseed rape fields. The vegetation indices with bigger contribution to detect flowers are NGRDI [14], Red Green Ratio Index (RGRI) [24], and Modified Green Red Vegetation Index (MGRVI) [25]. For classification, they used k-means applied on L*a*b color space. Random Forest [26] was also included to predict the flower number.

The authors in [4] proposed a spectral-spatial classifier based on a single hidden layer feed-forward neural network. The classifier uses RGB high-spatial-resolution images acquired from UAVs. The goal of the study was to detect, delineate, and count tree crowns. The input of the classifier was the RGB values of the pixels in the images, and the output was a binary response (tree or non-tree pixels). The authors carried out the spatial classification through thresholded geometrical property filtering techniques. The counting and delineation were done by means of the Watershed algorithm [27].

In [5], a system for the automatic quantification of wheat ear was proposed based on images acquired by an RGB conventional camera. The algorithm considers 3 steps: (1) detection of abrupt changes by means of Laplacian frequency filter [28], (2) median filtering to smooth the noise, and (3) a segmentation step using Find Maxima. The researchers developed an algorithm through the image analysis system ImageJ [29]. The algorithm achieved a success rate higher than 90% between the algorithm counts and the manual ear counts. The authors in [6] investigated a segmentation approach based on U-net [30] and a convolutional network net (CNN) [31], in combination with interpreted training data acquired by means of UAV-based high-resolution RGB imagery. The purpose of the research was to obtain a fine-grained mapping of vegetation species and communities. The authors in [6] achieved an accuracy of 84% and demonstrated that fusion between UAVs and artificial intelligence is applicable to a wide range of tasks in agriculture.

Mathematical morphology (MM) is also a powerful tool for discriminating a wide range of shapes and sizes in the image structure. MM is based on a set operators that transform images taking into consideration their topological and geometrical properties. Among the properties are the size of the regions and the shape. The most important operators are dilation, erosion, opening, and closing [8]. In [32], the researchers proposed an approach in which they analyze images acquired through UAV by means of MM in order to detect failures in coffee crops. The objective of the proposal was to evaluate product quality and the optimal occupation of planted areas. The researchers in [33] proposed a technique based on deep learning and computational vision using UAV images with the purpose of counting corn plants. Kitano and collaborators [33] used the opening morphological operator to separate detected objects after the segmentation done by means of the U-net architecture [30]. Fan et al. [34] implemented an algorithm that combines a neural network with morphological operations and the Watershed algorithm [27]. The goal of their research was to detect and count tobacco plants. In [32,33,34], the MM allowed the separation between objects of interest, removing of holes and protrusions, which is very important to preserve the main features of the detected objects and, later, to carry out any counting algorithm.

In [2], Calvario et al. proposed a methodology to discriminate agave plants using UAV, geomatics, and computer vision. The authors considered an UAV fixed flight to acquire agave plant images and the k-means algorithm for discriminating agave crops. Although this method has a good segmentation performance, it cannot be directly used for agave counting. Now, we propose a methodology for counting agave plants which is a continuation of the research done in [2]. Our proposal is based on the use of mathematical morphology. This strategy allows us to refine the solution provided in [2] and, at the same time, to obtain a good agave counting method. MM is a traditional image processing method [8,9] that does not require a training stage. For this kind of method, basically, hyperparameters are adjusted considering the features of the images of interest.

Artificial neural networks have recently gained a lot of attention due to the application of deep learning techniques allowing for obtainmentn of excellent results in different machine learning tasks [35]. The cost of obtaining such results is the increasing complexity of the underlying model. Therefore, this type of model requires large databases during the training step, and in general, they are not practical when the number of data is very low. In order to reduce this problem, some data augmentation has recently been studied. In the case of agave plants, the number of reported labeled images is very low, and to the best of our knowledge, there are no labeled agave databases that allow us to train deep learning data-based modeling. The selection of MM as a computer vision tool is also justified by the problem we face after the segmentation in [2]: the overlap between the detected agave plants, and the variation in size and shape. MM has a good performance to extract information about the shape and size, which are important features that we include in the proposal described in Section 3.

The structure of the manuscript in the following is as follows: Section 3 details the studied images and the proposal; the discussion about the obtained results is provided in Section 4; and finally, the conclusions are given in Section 5.

## 3. Materials and Methods

### 3.1. Study Areas

**Figure 3:**
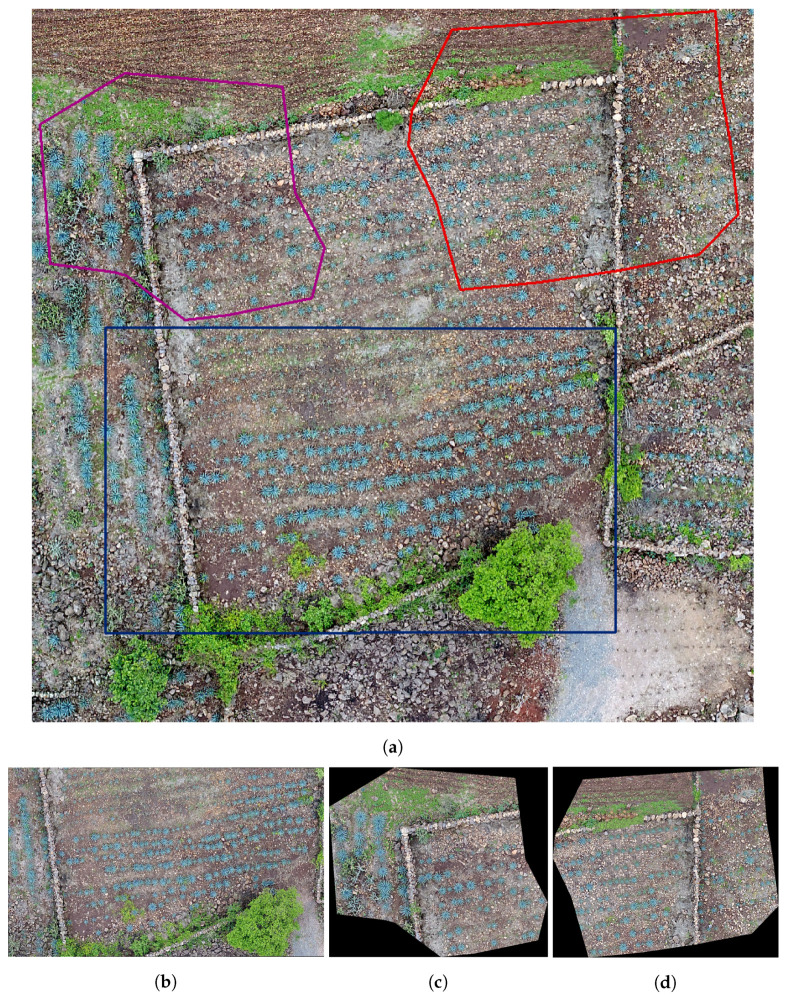
Images of Field 2 obtained with an UAV: (**a**) Field 2 with some marked regions that are studied in this work, (**b**) the region in blue, (**c**) the region in purple, and (**d**) the region in red.

**Figure 4:**
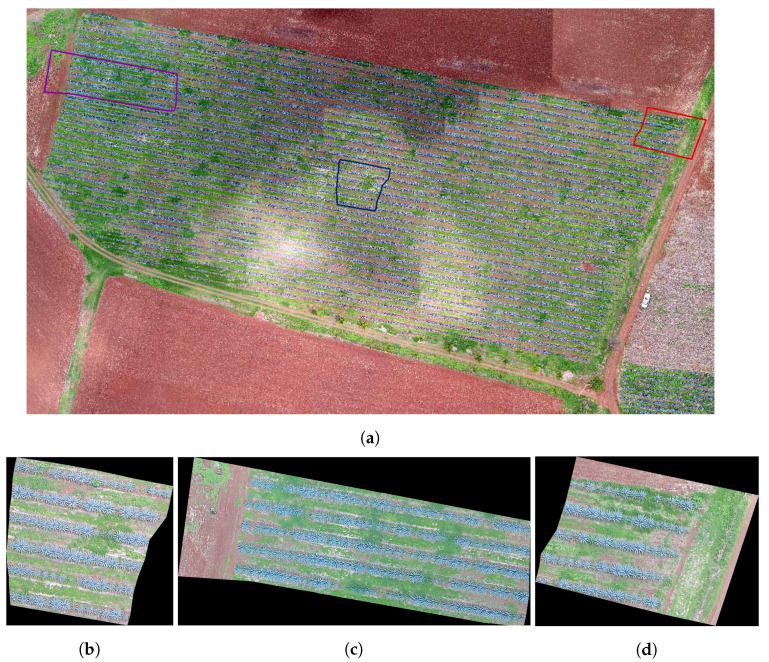
Images of Field 3 obtained with an UAV: (**a**) Field 3 with some marked regions that are studied in this work, (**b**) the region in blue, (**c**) the region in purple, and (**d**) the region in red.

The study looked at three different fields, with agave plants of different sizes, concentrations, and ages 0. Table 1 contains the geographical position, area, and age of agave plants for each studied field. Figure 2, Figure 3 and Figure 4 describe the features of each field.

Field 1 contains agave plants of different ages and sizes. Weed is another component of the field. Field 1 is of red clay type soil, and there is a color contrast between the plants and the soil. The overlap between plants is another important feature. Observe the areas represented by polygons in red, blue, and purple in Figure 2a–c. They demonstrate the above explanation.

Field 2 is of rocky soil, and there is less contrast between the plants and the soil compared to Field 1. Similar to Field 1, there are plants of different sizes, but the overlap problem is less with respect to Field 1.

Field 3 is of the same red clay as in Field 1. The overlap problem and weeds are also present. Note that, in this case, the variability of plant sizes is lower than the variability in Fields 1 and 2.

**Figure 5:**
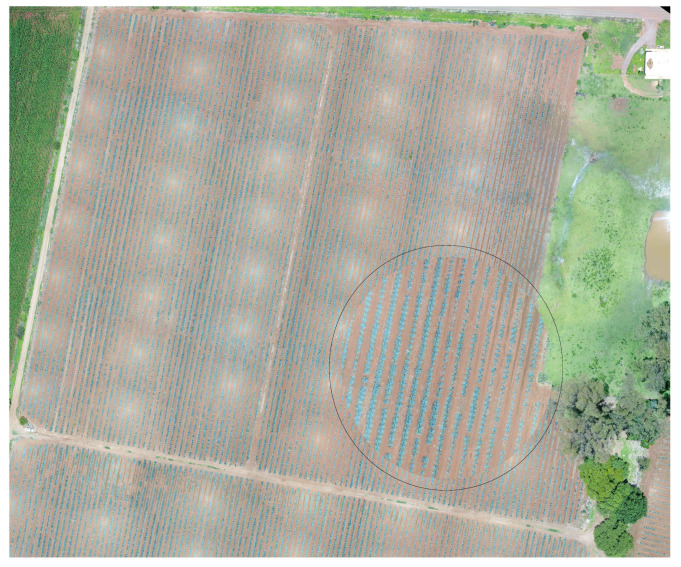
Agave plantation: the zone surrounded by a circle zooms in on the lines of the sowing system.

The sowing system of the blue agave is linear; see Figure 5. The lines can be made through a tractor, stakes, or manually with a thread. The furrows are distributed at a distance allowing for separation between the plant rows. Afterwards, a thread with marks is utilised to fix the needed distance between plants.

On average, the separation between each row is 3 to 4 m and the distance between plants is 1.00 to 1.20 m [36]. The diameter of the circle surrounding the plant width is about 1–2 m or more [37], so that one plant can be bigger than the distance between plants. This explains the overlap between plants.

### 3.2. Workflow

This work improves the segmentation result in [2] so that we can use it for agave counting purposes. Therefore, the input of our method corresponds to the output of the proposal in [2].

**Figure 6:**
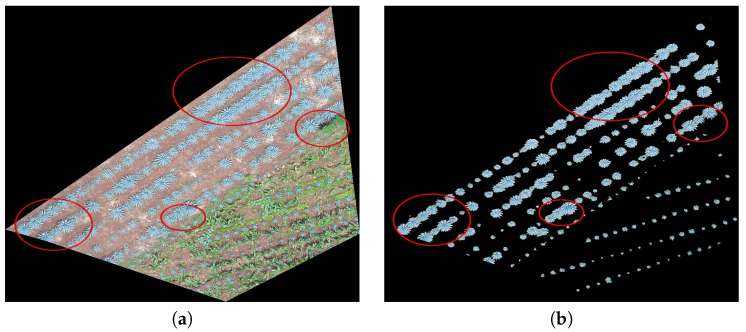
Extraction of the agave layer: the image in (**a**) represents the agave crops without applying the plant extraction methodology in [2]. The image in (**b**) shows the agave detection layer (segmented image) after applying the methodology. Circles in red highlight the overlapping zones.

Calvario et al. in [2] proposed a methodology based on photogrammetry and k-means algorithm. They use unmanned aerial vehicles of type Phantom 4, DJI, and the images are acquired with an RGB sensor with 6.25 mm × 4.68 mm and lens with a Field of View (FOV) of $94^{\circ }$ 20 mm. The spatial and bit resolutions of the image are 4000×3000 and 8 bit per pixel, respectively [2,38]. The UAV flight is planned taking into account the topography and Google Earth information. According to the experimental work, the selected flight altitude was 60 meters. In this work, we follow this recommendation; see the details in [2]. Once the images are acquired through the UAV, the photogrammetric processing is carried out. Following this, the authors in [2] generated the corresponding orthomosaic; see Figure 6a, with a spatial resolution of about 3 cm per pixel. The obtained image is converted from RGB color space to CIELab in order to use the Euclidean distance criterion in a perceptual uniform space. Subsequently, k-means was applied to discriminate agave plants from the rest of objects in the image. The detected classes, agave plants and background, are validated using the ground truth and geographic system information. For more details, see [2]. An example of a segmented image, obtained in [2], appears in Figure 6b. The final representation of the results is given in RGB.

Figure 6b depicts the input of our counting algorithm. Note the overlap problem in the segmentation results.

**Figure 7:**
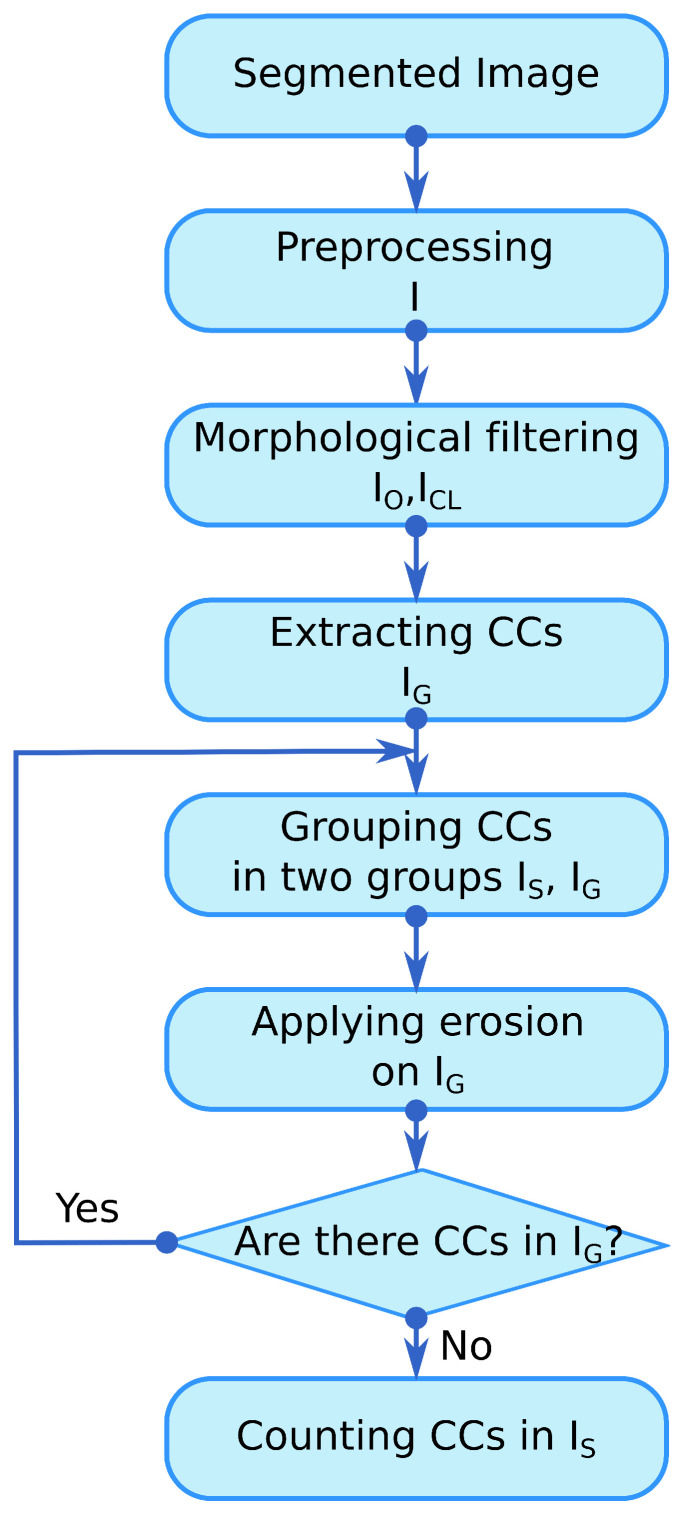
Flow chart of the implemented agave counting algorithm based on mathematical morphology: the abbreviation CC stands for connected component. The grouping step is carried out by a thresholding method. The segmented image is the output of the proposal in [2].

The graphical representation of the elaborated algorithm is depicted in Figure 7. A detailed explanation of the algorithm is given in Section 3.3.

### 3.3. Implementation of Mathematical Morphology Operations for Counting Agave Plants

The proposal is composed of 3 steps: preprocessing, object separation, and counting.

**Figure 8:**
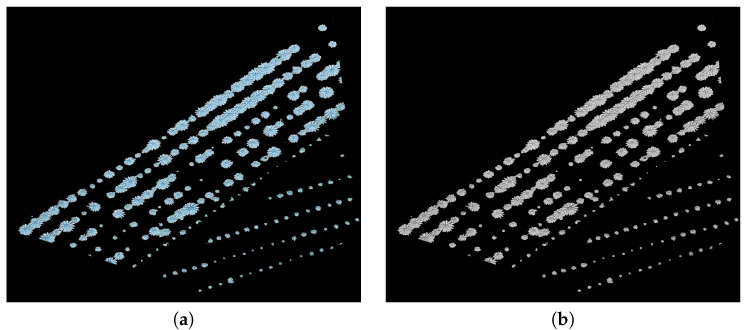
Preprocessing step: (**a**) a segmented image, the output of the work in [2], and (**b**) conversion from an RGB image to a gray-scale image.

**Figure 9:**
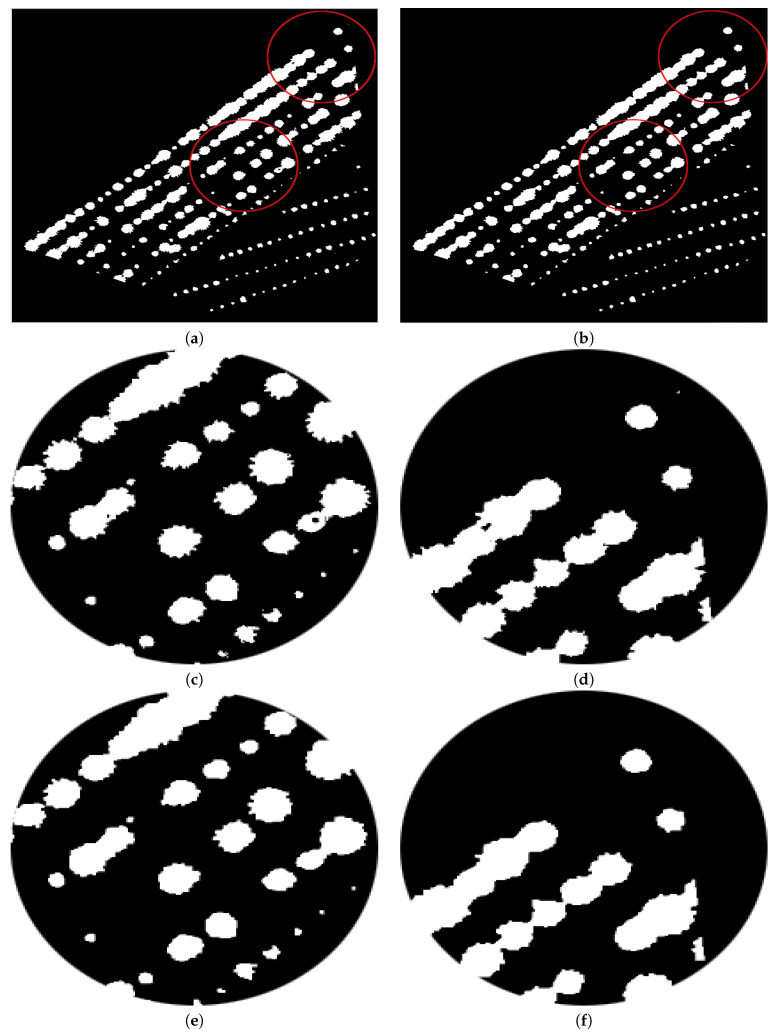
Preprocessing step: (**a**) binarization, (**b**) preprocessed image $I_{c}$, (**c**) enlarged image of the lower surrounded region in (**a**), (**d**) an enlarged image of the upper surrounded region in (**a**), (**e**) the result of removing and cleaning the region in panel (**c**), and (**f**) the result of removing and cleaning the region in panel (**d**).

**Figure 10:**
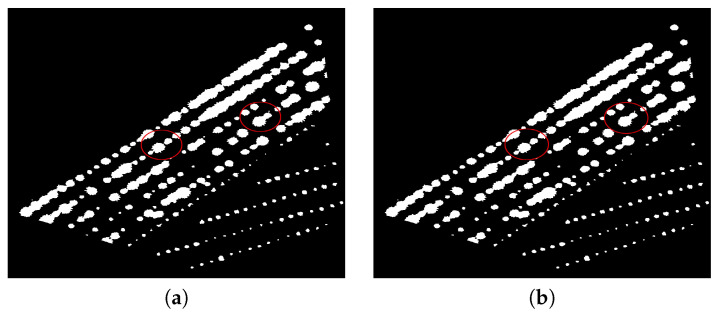
Results of applying opening and closing operators: (**a**) image obtained from opening and (**b**) closing morphological operators.

**Figure 11:**
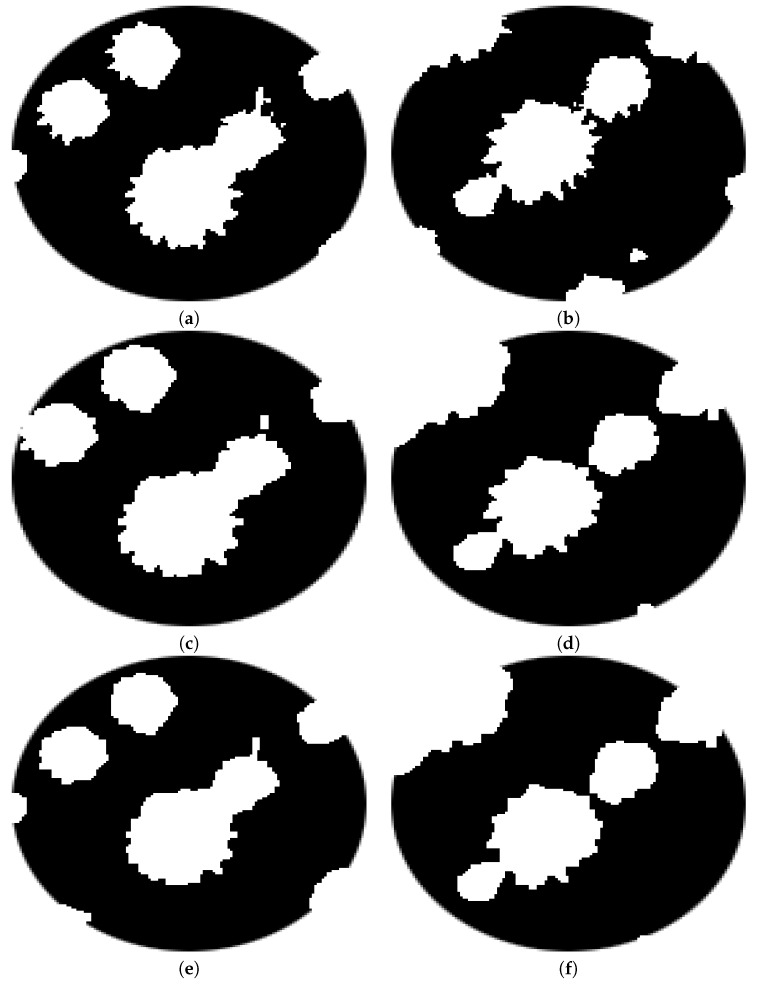
Morphological opening and closing: (**a**) zoom in on the circle in red on the left of Figure 10, (**c**) opening and (**e**) closing of the region in (**a**), (**b**) zoom in on the circle in red on the right of Figure 10, and (**d**) opening and (**f**) closing of the region in (**b**).

**Figure 12:**
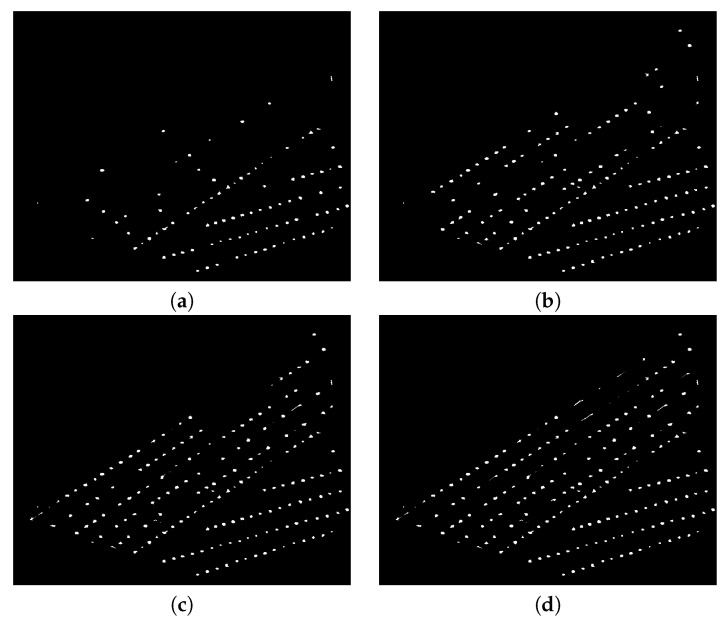
Images representing some iterations of the counting algorithm for a region in Figure 10: (**a**–**d**) show the detected plants after the iterations 1, 5, 8, and 13, respectively.

1. **Preprocessing**: The main aim of this step is to obtain a binary image as clean as possible so that we can achieve a good object separation in the next step. For this purpose, we carry out the following steps:
  - Firstly, we transform the segmented RGB color image into a gray-scale image by using the following equation:
    (1) I=0.299∗R+0.587∗G+0.114∗B
    where R,G, and *B* represent the red, green, and blue channels of the segmented image (Figure 8a) and *I* corresponds to the gray-scale representation (Figure 8b).
  - Secondly, we binarize the previous image *I* according to the following equation:
    (2) $$I\left(r\right)=\{\begin{array}{cc} 1 & I(r)>0\\ 0 & otherwise \end{array}$$
  - After the previous step, we typically achieve a noisy binary image with isolated points and holes. For this reason, we remove these artifacts in order to reduce false positives or false negatives during the counting process. For this step, we can use several image processing techniques. In particular, in this work, we use morphological filters, i.e., clean and fill morphological filters. We denote the final preprocessed image as $I_{c}$; see Figure 9. Note that, in this case, isolated points and holes are removed; compare the images in Figure 9c,d with the images in Figure 9e,f, respectively.
2. **Object separation**: The main objective of this step is to eliminate the overlap of regions corresponding to agave plants. However, this is a very hard task, so in practice, in this step, we reduce this problem. It is well known that morphological operators are efficient image-processing transformations that allows to attenuate the overlapping problem. Hence, in this step we apply a set of morphological opening and morphological closing operations [8,39]. One advantage of MM is that it allows to detect structures of different sizes and shapes. In the proposed algorithm, the rounded shape and diameter size are the features that define the patterns of agave plants to be counted. Following the recommendation of the Tequila Council in Jalisco Mexico, we need to preserve all patterns corresponding to each agave plant and its shoots. Therefore, the order of application of morphological operators to the images is very relevant. For the previous reason, morphological opening is firstly carried out:
  (3) $$I_{o}=\left(I_{c}⊖B\right)⊕B,$$
  where ⊖ and ⊕ represent the morphological erosion and dilation, respectively, [8,39]; *B* denotes the structuring element. In our case, we consider the squared structuring element of size 3×3.
  Afterwards, we apply morphological closing (see Equation (4)) with the goal of recovering any distorting pattern of the agave plant as a result of morphological opening.
  (4) $$I_{cl}=\left(I_{o}⊕B\right)⊖B$$
  $I_{cl}$ denotes the image after closing operation and *B* is the same squared structuring element used in Equation (3).
  Figure 10 and Figure 11 depict an example of morphological opening and closing steps.
3. **Counting**
  The counting process is an iterative procedure that combines the extraction of connected components with morphological operators. The stages of this procedure are described below:
  - **1**: Extracting all connected components.
  - **2**: Separating small connected components, $I_{S}$, from greater ones, $I_{G}$, through a threshold Th. $I_{S}$ and $I_{G}$ denote the images obtained after thresholding. Th was fixed through experiments, and it is given in pixels units. Th is related to the equivalent diameter: thte diameter of a circle which has the same area as the connected component to be considered in the counting process.
  - **3**: Morphological erosion of the image $I_{G}$ using a structuring element with a diamond shape of size 2, according to Equation (5):
    (5) $$I_{G}=I_{G}⊖B$$
    The purpose of the erosion filter is to separate in each iteration, as much as possible, the patterns corresponding to each individual plant. The erosion filter attenuates the overlap problem.
  - **4**: Finding all the connected components in the image $I_{G}$. If there is any connected component, repeat the process from **1** to **4**; otherwise, go to **5**.
  - **5**: Counting of all connected components saved in the image $I_{S}$, see Figure 12.
  The threshold value, Th, and the size of the structuring elements for the mathematical morphology are the parameters of our proposal. They were tuned experimentally with respect to a fixed flight altitude and the segmentation results reported in [2]. According to our experimentation, connected components with an equivalent diameter less than 13 pixels are candidates for representing patterns of individual agave plants. For that reason, Th is 13. The proposed counting algorithm was written in Matlab (R2016a).

### 3.4. Evaluation Metrics

To assess the performance of our proposal, we utilise four accuracy metrics which are based on the confusion matrix. Equations (6)–(9) detail about them [40,41]. In this manuscript, we use the same notation as the authors in [42,43].

The *producer’s accuracy* $P_{acc}$

(6) $$P_{acc}=\frac{TP}{GT}$$

can be interpreted as the percentage of the agave plants correctly identified. TP denotes the true positives in the confusion matrix and represents the number of agave plants correctly identified by the proposal and GT indicates the ground truth of agave plants in the image.

The *user’s accuracy* $U_{acc}$

(7) $$U_{acc}=\frac{TP}{TP+FP}$$

is a measure of the reliability of the agave plants layer and indicates the percentage of the agave class that corresponds to the ground truth agave plants. FP denotes false positive detection and represents the total number of agave plants incorrectly identified by the proposal.

The *recall* or true positive measure is given by the following equation:

(8) $$recall=\frac{TP}{TP+FN},$$

where FN denotes false negative detection and represents the number of agave plants that were not identified by the proposal.

The *average accuracy* measure, Acc,

(9) $$Acc=\frac{P_{acc}+U_{acc}}{2}$$

combines expressions (6) and (7); such an accuracy measure has the advantage of taking into account both the results of agave plants correctly identified and the number of false positives and refers to the average of the two previous accuracy measures.

## 4. Results and Discussion

The parameters of our proposal are the *threshold value*, Th, and the *size of the structuring elements* for mathematical morphology. These parameters are easy to establish, and they depend basically on the UAV flight altitude and the agave plant pattern. In our case, we tuned these parameters experimentally with respect to the fixed flight altitude and segmentation results reported in [2]. According to our experimentation, connected components with an equivalent diameter less than 13 pixels are candidates for representing patterns of individual agave plants. For this reason, we set Th=13 in the experiments. In order to find the shape and size of the structure element, we used the gridsearch strategy, which is a standard technique in statistics [44] and machine learning for hyperparameter optimization or tuning [45,46]. This technique is implemented in software packages such as LIBSVM—A Library for Support Vector Machines [47] and Scikit-learn Machine Learning in Python [48]. Details about the shape and size of the structure element are provided in Section 3.3.

For all given examples, a threshold value Th=13 pixels was used, which is equivalent to 39 centimeters, taking into account that, for the fixed flight altitude in [2], the ground sampling distance is approximately equal to 3 cm. The Th value was adjusted after many trials, and it was corroborated by real measures in the crop area. The size and the shape of the structuring element used in this proposal were also adjusted experimentally and by taking into account the results of the methodology in [2].

**Figure 13:**
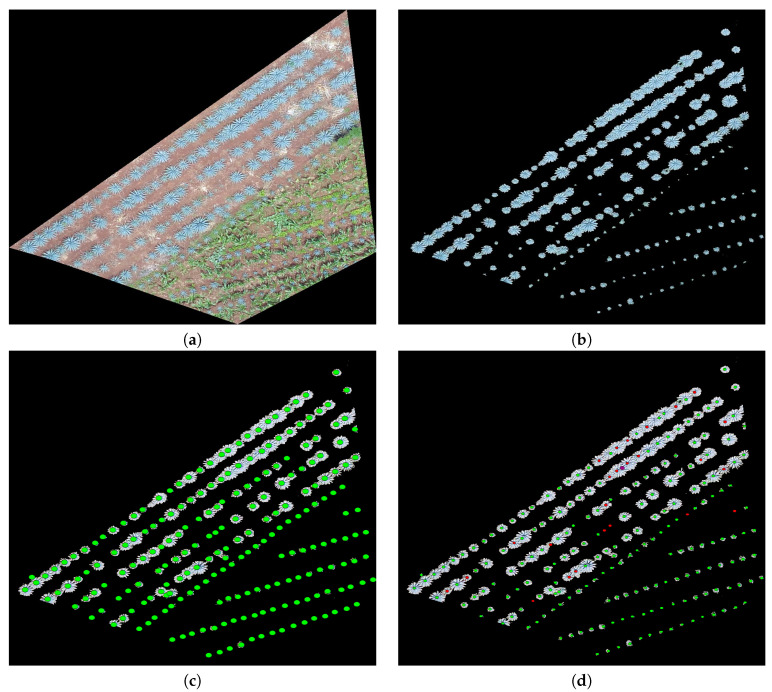
(**a**) A polygon in blue in Field 1 ( Figure 2), (**b**) an extracted agave layer by means of the methodology in [2], (**c**) agave plants counted by an expert (ground truth), and (**d**) agave plants detected by the proposal: those not detected appear in red (FN).

**Figure 14:**
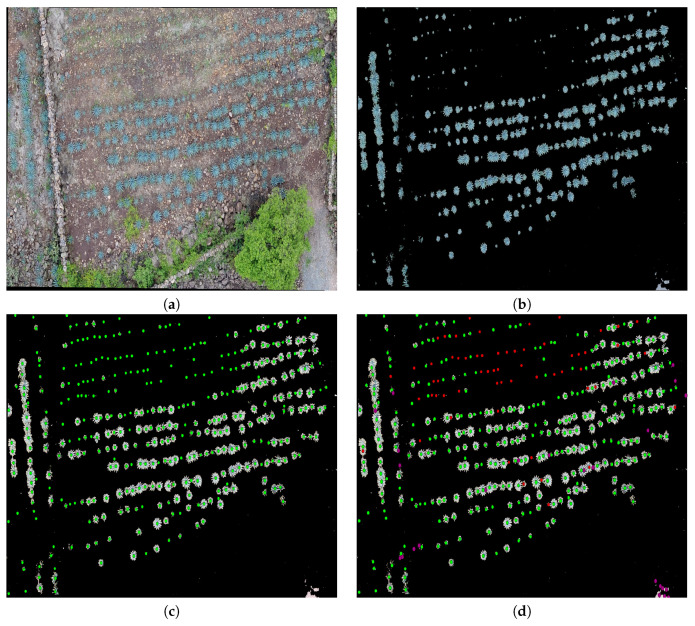
(**a**) A polygon in blue in Field 2 (Figure 3), (**b**) an extracted agave layer by means of the methodology in [2], (**c**) agave plants counted by an expert (ground truth), and (**d**) agave plants detected by the proposal: those not detected appear in red (false negative), while false positives appear in purple.

**Figure 15:**
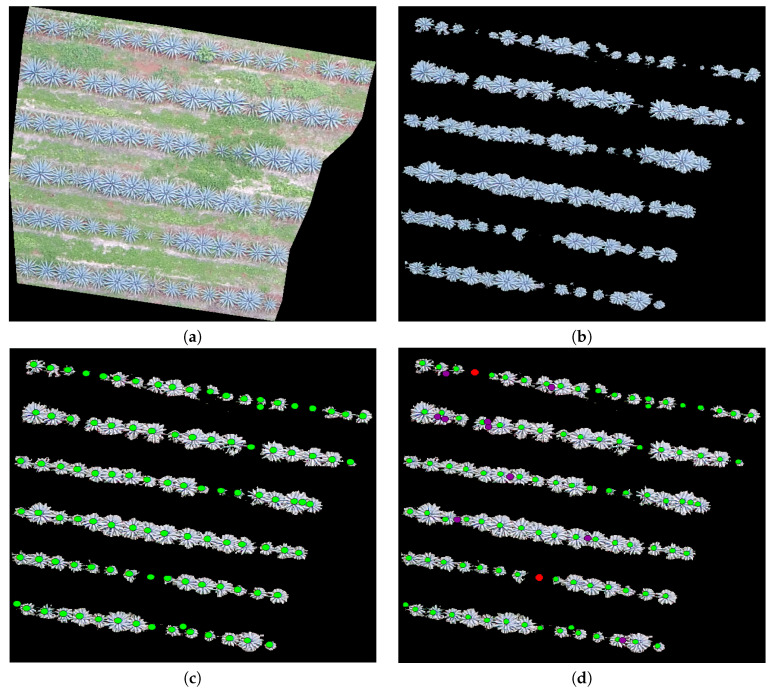
(**a**) A polygon in blue in Field 3 (Figure 4), (**b**) an extracted agave layer by means of the methodology in [2], (**c**) agave plants counted by an expert (ground truth), and (**d**) agave plants detected by the proposal; those not detected appear in red (false negative).

In the experiments, we apply the algorithm described in Section 3.3 to the highlighted regions in Figure 2, Figure 3 and Figure 4. The results can be seen in Figure 13, Figure 14 and Figure 15.

Figure 13c,d depicts the manual counting executed by an expert and by our proposal, respectively. Detected agave plants appear in green. Plants not detected by our proposal are indicated in red (FN); see Figure 13d. Notice that, for the image in Figure 13a, in almost all cases, the algorithm fails when the plants are overlapped (circles in red) or they are very small. They were not detected in the agave layer with the methodology provided in [2]. It should be noted that the proposal fails a little in reference to the lower area that is full of grass or brush; see Figure 13.

Unlike the example in Figure 13a, the segmented image given in Figure 14b through the methodology in [2] was affected by illumination problems. This fact is reflected by the results of our counting algorithm; see Figure 14d. In this case, we have not only false negatives but also false positives. False negatives appear in red, and false positives appear in purple. The counting done by an expert is illustrated in Figure 14c. Illumination problems in the given image affected the extraction of the agave plants, and as a consequence, it was impossible to count them all. This explains the false negative results when the proposal tries to detect very small plants. Further, the illumination problems bring about the false positive results; see the circles in purple in Figure 14d.

The results shown in Figure 15 are similar to those in Figure 13. Very small plants were not detected, and the overlap problem affected counting. False positives (a circle in purple) are artifacts in the image due to the acquisition process in [2].

The numerical results in Table 2 give information about the performance of the counting algorithm applied on 3 regions in Field 1, Field 2, and Field 3; see Figure 2, Figure 3 and Figure 4. In this table, the symbol *S* refers to the samples in Figure 2, Figure 3 and Figure 4, while GT denotes the number of agave plants detected by an expert, i.e., the ground truth. The numerical information corresponds to applying the accuracy metrics described in Section 3.4.

We observe (see column FN) that the number of false negatives in Field 1 is greater than in the rest of the fields. This is due mainly to the overlap problem and the variability in size and shape of plants in Field 1; see Figure 2. On the other hand, Field 3 has the least number of false negatives. In this field, the agave plants are more homogeneous in size and age. Although the overlap problem is also present, the area corresponding to each plant is more delimited in comparison with other two fields (Figure 3 and Figure 4. According to column FP in Table 2, the samples in Fields 2 and 3 have the highest false positives. In this case, the weed segmented as agave plants explains the number of FPs. From Table 2, it could be observed that the highest values of $P_{acc}$ were obtained for samples in Fields 2 and 3, which is in correspondence with the lowest FN values over these samples. The highest value of $U_{acc}$ corresponds to samples in Field 1 because this Field has the lowest number of FPs. The highest values of $A_{cc}$ and recall are achieved in samples of Fields 2 and 3. This is due to less variability in size and age and less overlap among the agave plants. The measure $A_{cc}$ achieved a value in the range from 0.8892 to 0.9594. Overall, the $A_{cc}$ for the nine considered regions is 0.8955.

The proposed counting algorithm is able to detect a wide range of agave plants in size and age. This proposal has been applied on plantations located in Jalisco State, Mexico, and was verified by the Tequila Regulatory Council (CRT) in order to promote precision agriculture in Mexico. According to CRT, the obtained results are satisfactory and contribute towards the improvement of agave crop monitoring and therefore a better production control of the tequila drink [49].

## 5. Conclusions and Future Work

The number of blue agave plants is an important parameter for estimating the yield of plantation and for planning the production of Tequila, a Mexican beverage obtained from agave. In this work, an agave counting algorithm was proposed in order to improve the monitoring of agave crop and to control the yield of the plantation in Jalisco, Mexico.

To the best of our knowledge, this is the first algorithm aimed at automatic counting of agave plants in Mexico.

The proposed counting algorithm is based on mathematical morphology. This is a standard image-processing technique very useful for separating and counting structures by their size and shape; this justifies our choice. The proposal combines the results of a segmentation algorithm with the application of a well-structured sequence of morphological operations so as to achieve a good plant separation and, accordingly, reliable counting. The use of mathematical morphology allows us to reduce the overlap and lighting issues that are not fully resolved in the segmentation step. The proposed algorithm is able to separate agave plants and to preserve the main agave plant patterns, obtaining a reliable count with a producer’s accuracy in a range of 0.8309 to 0.9806.

As feature work, we consider creating a database increasing the number of labeled agave plant images so that we can use supervised learning techniques like an artificial neural network and in particular deep learning techniques.

## Figures and Tables

**Table 1:** Geographical position and area of studied subregions.

Field	Geographical Position	Age	Area (ha)
1	$20^{\circ }$$34^{\prime }$32.22${}^{\prime \prime }$ N, $102^{\circ }$$25^{\prime }$10.06${}^{\prime \prime }$ W	2–4 years	2.494
2	$20^{\circ }$$44^{\prime }$34.49${}^{\prime \prime }$ N, $102^{\circ }$$27^{\prime }$30.14${}^{\prime \prime }$ W	4 years	0.4293
3	$20^{\circ }$$40^{\prime }$05.69${}^{\prime \prime }$ N, $102^{\circ }$$39^{\prime }$06.23${}^{\prime \prime }$ W	4 years	4.018

**Table 2:** Computed accuracy metrics.

*S*	*TP*	*FN*	*FP*	*GT*	*P_acc_*	*U_acc_*	*Recall*	*A_cc_*
Field 1-Blue	198	25	1	223	0.8879	0.9950	0.8979	0.9414
Field 1-Purple	336	33	9	369	0.9106	0.9739	0.9106	0.9422
Field 1-Red	344	70	11	414	0.8309	0.9690	0.9309	0.9000
Field 2-Blue	326	60	25	386	0.8446	0.9288	0.8446	0.8867
Field 2-Purple	99	6	13	105	0.9429	0.8839	0.9429	0.9134
Field 2-Red	177	8	7	185	0.9568	0.9620	0.9568	0.9594
Field 3-Blue	101	2	10	103	0.9806	0.9099	0.9806	0.9452
Field 3-Purple	192	7	25	199	0.9648	0.8848	0.9648	0.9248
Field 3-Red	78	5	15	83	0.9398	0.8387	0.9398	0.8892
